# The Administration of Chloroform

**Published:** 1869-06

**Authors:** 


### Selections.

THE ADMINISTRATION OF CHLOROFORM.—A. M. Rosebrugh, M. D., Surgeon to the Toronto Eye Dispensary, in a paper on "Chloroform and a new method of Administering it," states that he has been conducting a series of experiments with the object of determining the minimum quantity of chloroform necessary for inducing narcotism at different ages, and for different purposes, and to administer the chloroform in such a manner as to gain a pretty correct estimate of the degree of dilution of the vapor that is being administered at a given time. In this he has been successful. His method of administering the anæsthetic is as follows: the patient is placed on his back and a linen napkin placed over the face, so that one thickness only covers it. A two-drachm vial is filled with chloroform; an assistant observes the pulse, and holds the watch in such a position that the administrator may see the second-hand. The administrator assumes a convenient position at the head of the patient, and everything being ready, with the left hand he raises the napkin so that it does not touch the nose, about one and a half inches from the mouth. The chloroform is now carefully dropped upon the napkin over the mouth, a definite number of drops being allowed to fall per minute, commencing with a minimum quantity and gradually increasing until, in the third minute, the maximum quantity is reached. One-third the maximum dose is given during the first minute, and two-thirds during the second. The maximum dose should be continued from two to six minutes, according to the effect of the anæsthetic upon the patient, and the degree of narcotism desired. Where it is necessary to keep up the narcotism for a length of time, the maximum quantity of chloroform may be repeated occasionally—as often as the condition of the patient may seem to require—or about one-half the maximum quantity may be administered continuously. No attempt has hitherto been made to conduct the *guttatim* method so that, 1st. The administration shall commence with an almost imperceptible quantity of chloroform-vapor, and the strength be gradually increased as the system will tolerate it. 2. After tolerance is established, the administration shall continue with a certain definite quantity per minute until narcotism is established. 3d. The administrator shall be able to ascertain the percentage of chloroform-vapor that is being administered at a given time. In this he claims originality.

To adults, thirty drops per minute is sufficient in most cases. For children eleven or twelve years of age, a maximum quantity of eighteen drops per minute is sufficient. In all cases about one-third the maximum dose is given the first minute, and two-thirds the second minute; the maximum dose never being reached until the third minute from the commencement of the inhalation.

